# Supplementary material for: Economic burden of venous thromboembolism in surgical patients: A propensity score analysis from the national claims database in Vietnam
Source: PLoS One. 2020 Apr 9;15(4):e0231411. doi: 10.1371/journal.pone.0231411 (PMC7145013; doi:10.1371/journal.pone.0231411)
Supplement: Attachment — Submitted filename: Response for PONE-D-19-28967.docx [file pone.0231411.s001.docx]

Thank you for your feedback. Our responses and edits in blue as follow.

Journal Requirements:

1.When submitting your revision, we need you to address these additional requirements.

 Please ensure that your manuscript meets PLOS ONE's style requirements, including those for file naming. The PLOS ONE style templates can be found at

<http://www.journals.plos.org/plosone/s/file?id=wjVg/PLOSOne_formatting_sample_main_body.pdf> and <http://www.journals.plos.org/plosone/s/file?id=ba62/PLOSOne_formatting_sample_title_authors_affiliations.pdf>

Thank you very much for your comment. We have reviewed and edited it in our manuscript.

2. In ethics statement in the manuscript and in the online submission form, please provide additional information about the patient records used in your retrospective study. Specifically, please ensure that you have discussed whether all data were fully anonymized before you accessed them and/or whether the IRB or ethics committee waived the requirement for informed consent. If patients provided informed written consent to have data from their medical records used in research, please include this information.

Thank you very much for your comment. Our research was approved by the Ministry of Science and Technology in accordance with Decision No. 3622 and approved by the Ethics Committee of the Health Ministry No. 67 under the registry model, so all patients agree verbally and/or in the pre-surgical written consent, all consent to the use of medical data and information for the hospital’s training and research. All patients are anonymous and data collection and analysis are performed by many people.

3.  We noticed you have some minor occurrence of overlapping text with the following previous publication(s), which needs to be addressed:

<https://www.sciencedirect.com/science/article/pii/S2212109915000254?via%3Dihub>

<https://www.ncbi.nlm.nih.gov/pmc/articles/PMC2613997/>

In your revision ensure you cite all your sources (including your own works), and quote or rephrase any duplicated text outside the methods section. Further consideration is dependent on these concerns being addressed.

Thank you for your comments. We will edit according to your suggestion

4.

We note that you have indicated that data from this study are available upon request. PLOS only allows data to be available upon request if there are legal or ethical restrictions on sharing data publicly. For information on unacceptable data access restrictions, please see <http://journals.plos.org/plosone/s/data-availability#loc-unacceptable-data-access-restrictions>.

In your revised cover letter, please address the following prompts:

a) If there are ethical or legal restrictions on sharing a de-identified data set, please explain them in detail (e.g., data contain potentially identifying or sensitive patient information) and who has imposed them (e.g., an ethics committee). Please also provide contact information for a data access committee, ethics committee, or other institutional body to which data requests may be sent.

Thank you very much for your concern. The Vietnam law requirement of our research is that data sharing is not permitted, as our data set is large, related to patients’ information which belong to hospital and national insurance, and its related to national security. The Ethics Committee and Health Ministry did not allow any activities related to data sharing in or outside the country.

b) If there are no restrictions, please upload the minimal anonymized data set necessary to replicate your study findings as either Supporting Information files or to a stable, public repository and provide us with the relevant URLs, DOIs, or accession numbers. Please see <http://www.bmj.com/content/340/bmj.c181.long> for guidelines on how to de-identify and prepare clinical data for publication. For a list of acceptable repositories, please see <http://journals.plos.org/plosone/s/data-availability#loc-recommended-repositories>.

[Note: HTML markup is below. Please do not edit.]

Reviewers' comments:

Thank you for your comments. As we said above, as our data set is large, contain patients’ personal information and it related to national security. Thus, data sharing is not permitted. We are very sorry for this inconvenience.
Reviewer's Responses to Questions

**Comments to the Author**

1. Is the manuscript technically sound, and do the data support the conclusions?

The manuscript must describe a technically sound piece of scientific research with data that supports the conclusions. Experiments must have been conducted rigorously, with appropriate controls, replication, and sample sizes. The conclusions must be drawn appropriately based on the data presented.

Reviewer #1: No

Reviewer #2: Partly

Reviewer #3: Yes

Reviewer #4: Partly

2. Has the statistical analysis been performed appropriately and rigorously?

Reviewer #1: Yes

Reviewer #2: Yes

Reviewer #3: Yes

Reviewer #4: I Don't Know

3. Have the authors made all data underlying the findings in their manuscript fully available?

The [PLOS Data policy](http://www.plosone.org/static/policies.action#sharing) requires authors to make all data underlying the findings described in their manuscript fully available without restriction, with rare exception (please refer to the Data Availability Statement in the manuscript PDF file). The data should be provided as part of the manuscript or its supporting information, or deposited to a public repository. For example, in addition to summary statistics, the data points behind means, medians and variance measures should be available. If there are restrictions on publicly sharing data—e.g. participant privacy or use of data from a third party—those must be specified.

Reviewer #1: Yes

Reviewer #2: Yes

Reviewer #3: Yes

Reviewer #4: Yes

4. Is the manuscript presented in an intelligible fashion and written in standard English?

PLOS ONE does not copyedit accepted manuscripts, so the language in submitted articles must be clear, correct, and unambiguous. Any typographical or grammatical errors should be corrected at revision, so please note any specific errors here.

Reviewer #1: Yes

Reviewer #2: Yes

Reviewer #3: No

Reviewer #4: No

5. Review Comments to the Author

Please use the space provided to explain your answers to the questions above. You may also include additional comments for the author, including concerns about dual publication, research ethics, or publication ethics. (Please upload your review as an attachment if it exceeds 20,000 characters)

Reviewer #1: I have the following comments about this paper:

1. My main difficulty is that the authors have combined portal vein thrombosis (PVT) with DVT, PE and thrombophlebitis. PVT is often associated with general surgical problems and is caused by intra-abdominal inflammatory conditions, intra-abdominal sepsis, splenectomy, etc and this state is clearly different than DVT and PE. PVT is not considered one of the forms of VTE, which is usually DVT and PE. The authors should remove PVT and PTP (phlebitis and thrombophlebitis) from the VTE category and talk about them separately in the abstract and conlcusions.

Thank you for your comments. We have reviewed our manuscript and removed PVT and PTP from the VTE category, and supplement the number of patients who both have DVT and PE

2. Why would the authors exclude any anticoagulant treatment during the hospital admission? I would assume that most of the patients they are looking at would have received some type of DVT prophylaxis, so I would think this would have excluded most patients?

Reviewer #2: These results (other than the actual cost data for your country) are well known. What is the generalizable "new" message for the literature?
You did not mention VTE prophylaxis. What percent of patients with VTE received prophylaxis? Did you control for that in your propensity model>

Thank you very much for your comments. This is the first study in Vietnam to assess the direct economic burden of VTE in patients after surgery. This burden is significant compared to the GDP of Vietnamese’s income and it can be prevented

We tried to put VTE prophylaxis into our PSM model but the results did not match, so we temporarily removed it and will report in our next study about “Cost-effectiveness analysis of VTE prophylaxis in surgical patient” as soon as possible.

Reviewer #3: 1. General comments
This study used a Vietnamese national healthcare insurance database in order to evaluate the incidence and costs associated with venous thromboembolism (VTE) in post-surgical patients over an almost 2 year period.

The methods of this paper are strong. Specifically, the use of propensity score matching is one of the major strengths of the paper. Furthermore, the results are compelling, and of interest to the target audience. Nonetheless, the paper is poorly written and difficult to follow in its current form.
I believe that the manuscript will be worthy of publication once it is thoroughly revised.

Thank you very much for your comments. We have edited our manuscript according to your contributions.

2. Abstract:
a. No specific comments beyond that detailed below for each section.

3. Introduction:
a. The introduction section would benefit from some grammar and sytax editing, but otherwise I have no major concerns regarding the introduction section. I would recommend including one additional comment regarding the incidence of VTE in post-operative patients who do receive chemical thromboprophylaxis, in addition to the single statement about incidence in patients who do not receive chemical prophylaxis, since most hospitalized post-op patients do receive some form of thromboprophylaxis.

Thank you so much for your suggestion. We have reviewed and corrected our article in accordance with your comments.

4. Methods:
a. In the ‘Matched Controls’ section, the authors refer to Table 2 when discussing the Elixhauser Comorbidity Index, however Table 2 is unrelated to this statement.

Thank you for your comment. We have reviewed and correct it.
5. Results:
a. Table 2 needs to be explained more clearly, and also needs to be discussed in the main text of the article. Currently, the only reference to Table 2 (in the Methods section, as I described above) is inaccurate.

Thank you for your comment. We will add explanation for Table 2 more detail, and add it’s discussion in our manuscript. We will correct the inaccuracy related to reference of Table 2.

b. There are many acronyms used throughout the results section that are not explained at any point – RMPO, VET, OVP, PTP, etc

Thank you very much for your comment. We will add explanation for above acronyms into our manuscript.

c. Swap the order of mean ages in VTE vs. non-VTE group [sentence should read: “VTE group had higher rate of women and mean age than non-VTE group (50.9% vs. 40.2% and 60.5 year-old vs 45.7 year-old), as shown in Table 1]

Thank you very much for your comment. We have reviewed and edited according to your suggestion.

d. Surgery is spelled incorrectly in the first column of Table 1 and in the title for Table 2.

Thank you very much for your comment. We have reviewed and edited according to your suggestion

6. Discussion:
a. Similar to the introduction section, the discussion section requires significant revisions to grammar and syntax, but otherwise no major concerns.

Thank you for your comments. We will check and edit our manuscript

7. Figures and Tables:
a. Include a legend for each table, which includes what the acronyms stand for.
b. Refer to Table 2 somewhere in the main text (and delete the incorrect reference to Table 2 in the Methods section)

Thank you for your comments. We will check and edit our manuscript

8. Conclusion:
a. No comments

9. References:
a. No comments

Reviewer #4: In this paper, the authors want to understand the economic burden caused by Venous Thromboembolism (VET) in Vietnamese patients. They use a propensity score matching to identify a control group of patients with similar observable characteristic who did not suffer VET within 90 of hospital admission and compare them to a set of patients that did. Using this technique, they find that the cost of VTE within 90 days of the hospital visit was a little over $1,000.

In full disclosure, I am not a medical expert and so the specific medical mechanisms tested in the paper are very new to me. A medical researcher should take a much more careful look at the medical and public health claims they made. However, I have researched in Vietnam for a long time and have some expertise in the econometric techniques they are using to estimate their effects. Because I am outside the specific field of the researchers, there may be different norms in statistical presentation. This should be taken into account in in assessing my review.

I think there is a lot to admire in this paper. The problem is compelling and the authors have assembled an excellent data for their analysis. I also think the wide variety of outcome variables studied provides a thorough picture of the problem. However, there is a lot that needs improvement in this paper before publication. In particular, the paper does not meet current standard for the presentation of econometric analysis. While medical research may be slightly different, the economic conventions require a great deal more information to make sense of the results.

1. The matching strategy is not well described. What specific type of matching estimator was used (propensity score, genetic, strata, entropy balancing? Did the authors employ weights)? Did the match one-to-one or use multiple nearest neighbors? In fact, the authors don’t even show the matching estimation results in a table or appendix for the review to see. It is very hard to adjudicate the results the authors present without this information.

Thank you very much for your contributions.

We have collected information about the patient's weight index, but many patients are missing this index because weight measure was usually not performed during surgery time, resulting in heterogeneous in data set, so to avoid encountering bias when performing propensity score matching, we decide to exclude this parameter. Each patient in the venous thromboembolism (VTE) group is matched with 1 patient without VTE - or any other complications after surgery (within 90 days) using the trend score method. First, each patient will be estimated trend score by probit regression method, then using nearest neighbor 1: 1 matching method with patients with similar trend points. These steps are performed by psmatch2 in STATA 14.2 (E. Leuven and B. Sianesi. (2003). "PSMATCH2: Stata module to perform full Mahalanobis and propensity score matching, common support graphing, and covariate imbalance testing". Http: //ideas.repec.org/c/boc/bocode/s432001.html.). All patients with VTE were retained in the matching model with the no replacement option. In fact, our research team matched 1689 VTE patients with 1689 patients without VTE or any other postoperative complications (from 815,006 patients). Factors that have been included in the model to estimate trend scores are: gender, age group, surgery type, 6 ecological regions, Caprini score level comorbidities – according to Elixhauser et al

2. The key problem with matching strategies of all types is that one can only match on observables, when unobserved factors can drive both the presence of VET and the economic outcomes. The authors should make clear in their analysis what the potential threat from unobservable and what the potential direction of this bias is. In particular, I was concerned about whether VET is more likely in patients with particular baseline health profiles that are not picked up by the blunt covariates used in the match.

Thank you so much for your comments. The propensity matching score method can only partially solve the problem but cannot replace. Randomized clinical trials of the prospective study could control all confounding factors if the sample size was large enough. The P-score matching method in this study makes the most of possible variables to control confounding. Other factors will need further studies

3. Table 3 is not well described enough to understand what is going on. Is this a logit analysis based on the matched sample or between the VET sample and the RAW population? Are these the odd ratios generated for each covariate from a logit model. I cannot tell because the table does not provide the sample size? Why is only statistical significance given and not the standard errors? Finally no log-likelihood, chi-squared, or any measure of model fit is given in the table.

Thank you very much for your comment. We will review and edit our manuscript according to your suggestion.

4. Most importantly, it is highly unorthodox to control for covariates that were already included in the matching equation again as covariates in the estimation. What is the justification for this decision? If the authors are comparing to the matched sample, the covariates should be orthogonal to treatment by design and therefore irrelevant as control variables. This can lead to bias in the second stage.

Thank you so much for your comment. The main purpose of the comparison between the two groups is to show the homogeneity between the two groups after matching, not to look for any differences.

5. Table 4 is even more confusingly described. Are those standard errors parentheses? Are the authors comparing to the matched sample again? Again, we can’t tell because no n is provided. Why are different covariates selected from those used in Table 3? What justifies this choice? Why are no estimates of model fit presented?

Thank you so much for your comment. The Standard Error is written in parentheses, at there we are not comparing the two groups, we are comparing the difference between the two groups with 0. You can see in the method section, we have estimated the burden by taking the cost difference of a VTE patient compared to a matched patient from a non-VTE group. In more detail, we will only keep patients who have been matched from PSM, then split into two datasets and merge together using a common ID variable created by the psmatch2 command! Then the cost difference will be calculated

6. This statement is clearly biased upward by selection bias. It should not be the main finding presented in the abstract and conclusion. The matched estimates are half the size. “The 90-day cost of VTE patients was found to be US$2,913. There was a 5.4-fold increase in the rate of re-admission, 1.8-fold increase in the rate of outpatient visits and a 2.2- fold increase in total costs over 90 days in patients with VTE undergoing surgery.” The authors can put that in a footnote, but only the matched estimates should be used in presenting the size of the effects. They are the scientifically valid result.

Thank you for your comment and suggestion. We will check and correct it

7. I found the comparison to other countries in the discussion to be incomplete. Rather than providing the unadjusted estimates of VET costs for the US, Japan, and Europe, wouldn’t it make more sense to standardize by the cost of living in those countries (or at least by GDP per capita) to get a better sense of the true societal costs of the procedure.

Thank you so much for your suggestion. We will check and re-correct it

8. Relatedly, to make sense of the costs of VET in the Vietnam context, it would be helpful to compare the cost of VET to the projected costs of prevention. This could be weighted by the probability of VET in each surgery type to get a sense of what the Vietnamese medical system should do to avoid the costs.

All in all, the authors have done important work. However more work on statistical presentation is necessary to adjudicate the findings and ultimately for the work to be understood and acted upon by a broad audience.

Thank you very much for your comment

6. PLOS authors have the option to publish the peer review history of their article ([what does this mean?](https://journals.plos.org/plosone/s/editorial-and-peer-review-process#loc-peer-review-history)). If published, this will include your full peer review and any attached files.


If you choose “no”, your identity will remain anonymous but your review may still be made public.


**Do you want your identity to be public for this peer review?** For information about this choice, including consent withdrawal, please see our [Privacy Policy](https://www.plos.org/privacy-policy).

Reviewer #1: No

Reviewer #2: No

Reviewer #3: No

Reviewer #4: No


[NOTE: If reviewer comments were submitted as an attachment file, they will be attached to this email and accessible via the submission site. Please log into your account, locate the manuscript record, and check for the action link "View Attachments". If this link does not appear, there are no attachment files to be viewed.]


While revising your submission, please upload your figure files to the Preflight Analysis and Conversion Engine (PACE) digital diagnostic tool, <https://pacev2.apexcovantage.com/>. PACE helps ensure that figures meet PLOS requirements. To use PACE, you must first register as a user. Registration is free. Then, login and navigate to the UPLOAD tab, where you will find detailed instructions on how to use the tool. If you encounter any issues or have any questions when using PACE, please email us at [figures@plos.org](mailto:figures@plos.org). Please note that Supporting Information files do not need this step.
