# Supplementary material for: Economic burden of venous thromboembolism in surgical patients: A propensity score analysis from the national claims database in Vietnam
Source: PLoS One. 2020 Apr 9;15(4):e0231411. doi: 10.1371/journal.pone.0231411 (PMC7145013; doi:10.1371/journal.pone.0231411)
Supplement: Attachment — Submitted filename: Response to Reviewers.docx [file pone.0231411.s002.docx]

Thank you for your feedback. Our responses and edits in blue as follow.

5. Review Comments to the Author

Please use the space provided to explain your answers to the questions above. You may also include additional comments for the author, including concerns about dual publication, research ethics, or publication ethics. (Please upload your review as an attachment if it exceeds 20,000 characters)

Reviewer #1: I have the following comments about this paper:

1. My main difficulty is that the authors have combined portal vein thrombosis (PVT) with DVT, PE and thrombophlebitis. PVT is often associated with general surgical problems and is caused by intra-abdominal inflammatory conditions, intra-abdominal sepsis, splenectomy, etc and this state is clearly different than DVT and PE. PVT is not considered one of the forms of VTE, which is usually DVT and PE. The authors should remove PVT and PTP (phlebitis and thrombophlebitis) from the VTE category and talk about them separately in the abstract and conlcusions.

Thank you for your comments. We have reviewed our manuscript and removed PVT and PTP from the VTE category, and supplement the number of patients who both have DVT and PE

2. Why would the authors exclude any anticoagulant treatment during the hospital admission? I would assume that most of the patients they are looking at would have received some type of DVT prophylaxis, so I would think this would have excluded most patients?

Reviewer #2: These results (other than the actual cost data for your country) are well known. What is the generalizable "new" message for the literature?
You did not mention VTE prophylaxis. What percent of patients with VTE received prophylaxis? Did you control for that in your propensity model>

Thank you very much for your comments. This is the first study in Vietnam to assess the direct economic burden of VTE in patients after surgery. This burden is significant compared to the GDP of Vietnamese’s income and it can be prevented

We tried to put VTE prophylaxis into our PSM model but the results did not match, so we temporarily removed it and will report in our next study about “Cost-effectiveness analysis of VTE prophylaxis in surgical patient” as soon as possible.

Reviewer #3: 1. General comments
This study used a Vietnamese national healthcare insurance database in order to evaluate the incidence and costs associated with venous thromboembolism (VTE) in post-surgical patients over an almost 2 year period.

The methods of this paper are strong. Specifically, the use of propensity score matching is one of the major strengths of the paper. Furthermore, the results are compelling, and of interest to the target audience. Nonetheless, the paper is poorly written and difficult to follow in its current form.

I believe that the manuscript will be worthy of publication once it is thoroughly revised.

Thank you very much for your comments. We have edited our manuscript according to your contributions.

2. Abstract:

a. No specific comments beyond that detailed below for each section.

3. Introduction:

a. The introduction section would benefit from some grammar and sytax editing, but otherwise I have no major concerns regarding the introduction section. I would recommend including one additional comment regarding the incidence of VTE in post-operative patients who do receive chemical thromboprophylaxis, in addition to the single statement about incidence in patients who do not receive chemical prophylaxis, since most hospitalized post-op patients do receive some form of thromboprophylaxis.

Thank you so much for your suggestion. We have reviewed and corrected our article in accordance with your comments.

We have added in the introduction (p4):

*A research in knee arthroplasty operation Asian patients with and without pharmacological prophylaxis suggested that who were administered chemoprophylaxis did not have a statistically significant difference in incidence of VTE although it may indicate better function [9].*

4. Methods:
a. In the ‘Matched Controls’ section, the authors refer to Table 2 when discussing the Elixhauser Comorbidity Index, however Table 2 is unrelated to this statement.

It was a very regrettable mistake. Elixhauser’s set of 30 comorbidities was presented in Table 1.

5. Results
a. Table 2 needs to be explained more clearly, and also needs to be discussed in the main text of the article. Currently, the only reference to Table 2 (in the Methods section, as I described above) is inaccurate.

Thank you for your comment. We will add explanation for Table 2 more detail (p14), and add it’s discussion in our manuscript. We correctted the inaccuracy related to reference of Table 2.

*Table 2 presents the unadjusted cost and outcome of VTE and non-VTE group. The average 90-day surgery-related costs were $2,939 ± $3,834 for patients with VTE and $1,308 ± $1,923 for without VTE. The VTE patients who had come back to the hospitals for outpatient visits were 75.0% while this rate in non-VTE patients was 47.64%. Therefore, the cost of 90-day surgery-related outpatient visit of VTE patients were approximately 1.5-fold higher than the cost of 90 day surgery-related visits for non-VTE patients ($217.4 vs. $142.5). Notably, the readmission rate of VTE patients reached 43.92% which was rather high compared with 8.0% of non-VTE and the former’s drug costs was three-time as much as the latter’s ($455 vs. $141).*

b. There are many acronyms used throughout the results section that are not explained at any point – RMPO, VET, OVP, PTP, etc

Thank you very much for your comment. We will add explanation for above acronyms into our manuscript. You can check at the bottom of table and at the end of manuscript.

c. Swap the order of mean ages in VTE vs. non-VTE group [sentence should read: “VTE group had higher rate of women and mean age than non-VTE group (50.9% vs. 40.2% and 60.5 year-old vs 45.7 year-old), as shown in Table 1]

Thank you very much for your comment. We have reviewed and edited according to your suggestion.

*The VTE group had higher rate in women and mean age than the non-VTE group (52.17% vs. 47.83% and 61.1 year-old vs. 45.7 year-old), as shown in Table 1. (p9)*

d. Surgery is spelled incorrectly in the first column of Table 1 and in the title for Table 2.

Thank you very much for your comment. We have reviewed and edited according to your suggestion

6. Discussion:
a. Similar to the introduction section, the discussion section requires significant revisions to grammar and syntax, but otherwise no major concerns.

Thank you for your comments. We have checked and edited it in our manuscript

7. Figures and Tables:
a. Include a legend for each table, which includes what the acronyms stand for.
b. Refer to Table 2 somewhere in the main text (and delete the incorrect reference to Table 2 in the Methods section)

A legend of acronyms for each table has been added. We also corrected the problem in the Methods

*Moreover, we also used 30 chronic conditions derived from the chronic conditions developed by Elixhauser et al. (Table 1) to adjust risk and match VTE cases [16].*

8. Conclusion:
a. No comments

9. References:
a. No comments

Reviewer #3: The authors did not address any of the comments that I made in my initial review. I had major concerns about the manuscript as it was initially submitted, and since the authors did not address any of those concerns, I am not recommending that the manuscript be rejected.

We highly appreciate the comments of Reviewer#3. In order to correct all grammar and syntax errors as well as present the research in standard English, we have re-written almost parts of the article included Abstract, Introduction, Results and Discussions. We also add acronyms legends for tables. We found the mistake that you pointed in reference for Table 2 which actually belonged to Table 1. We also need to change the number in Tables as the requirement of Reviewer#1 so the Track changes file looks quite complicated. Maybe there are too many changes which made us difficult to show it in the Response to Reviewers. Thank you for your opinions and your patience. Please help us make the article better.
